# Trends in Roll-Your-Own Smoking: Findings from the ITC Four-Country Survey (2002–2008)

**DOI:** 10.1155/2012/406283

**Published:** 2012-05-13

**Authors:** David Young, Hua-Hie Yong, Ron Borland, Lion Shahab, David Hammond, K. Michael Cummings, Nick Wilson

**Affiliations:** ^1^Tobacco Control Unit, Cancer Council Victoria, 100 Drummond Street Carlton, VIC 3053, Australia; ^2^Cancer Research UK, Health Behaviour Unit, Department of Epidemiology and Public Health, University College, London WC1E6XA, UK; ^3^Department of Psychology, University of Waterloo, 200 University Avenue West, Waterloo, ON, Canada N2L 3G1; ^4^Department of Health Behavior, Roswell Park Cancer Institute, Elm and Carlton Streets, Buffalo, NY 14263, USA; ^5^Department of Public Health, University of Otago Wellington, P.O. Box 7343, Wellington South 6242, New Zealand

## Abstract

*Objective*. To establish the trends in prevalence, and correlates, of roll-your-own (RYO) use in Canada, USA, UK and Australia, 2002–2008. *Methods*. Participants were 19,456 cigarette smokers interviewed during the longitudinal International Tobacco Control (ITC) Four-Country Survey in Canada, USA, UK, and Australia. *Results*. “Predominant” RYO use (i.e., >50% of cigarettes smoked) increased significantly in the UK and USA as a proportion of all cigarette use (both *P* < .001) and in all countries as a proportion of any RYO use (all *P* < .010). Younger, financially stressed smokers are disproportionately contributing to “some” use (i.e., ≤50% of cigarettes smoked). Relative cost was the major reason given for using RYO, and predominant RYO use is consistently and significantly associated with low income. *Conclusions*. RYO market trends reflect the price advantages accruing to RYO (a product of favourable taxation regimes in some jurisdictions reinforced by the enhanced control over the amount of tobacco used), especially following the impact of the Global Financial Crisis; the availability of competing low-cost alternatives to RYO; accessibility of duty-free RYO tobacco; and tobacco industry niche marketing strategies. If policy makers want to ensure that the RYO option does not inhibit the fight to end the tobacco epidemic, especially amongst the disadvantaged, they need to reduce the price advantage, target additional health messages at (young) RYO users, and challenge niche marketing of RYO by the industry.

## 1. Introduction

This paper explores patterns of roll-your-own (RYO) use in four developed countries (USA, UK, Canada, and Australia). RYO cigarettes are an important component of the tobacco market in many countries, with wide variation in use. For example, a majority of smokers use RYO at least some of the time in New Zealand (NZ) (53%) [1] and Thailand (58%) [2], compared with 7% in the USA [3]. In 2002, the other three countries in the study reported here had intermediate prevalence with 28% in UK, 24% in Australia, and 12% in Canada [3].

The 2002 cross-sectional study [3] found that RYO use was associated with lower income, male sex, greater nicotine addiction, lower intention to quit, and greater likelihood to believe RYO tobacco is less harmful to health. In NZ [1] there was a strong interaction between age and socioeconomic status (SES), with use amongst younger smokers increasing more as SES declined, relative to older smokers, suggesting uptake of RYO is a strategy of younger, poorer smokers. SES is also important in middle income countries; in Malaysia and Thailand RYO smoking was associated with low income, low education, and being unemployed [2].

The primary driver for RYO is the price differential between factory-made (FM) and RYO cigarettes, due in part to differences in how these products are taxed [1, 2]. Not only is RYO tobacco subject to lower taxation in many countries, but it is also much easier to control the amount of tobacco used by rolling thinner cigarettes [4]. Evidence from previous ITC Project RYO studies [3, 5] also indicates that RYO smokers have a disproportionate tendency to believe RYO tobacco is less harmful and 20–30% cite “it (RYO) is not as bad for your health” as a reason for smoking RYO [1, 6], even though research suggests that RYO cigarettes are at least as harmful, and if anything more harmful, than FM cigarettes [7–10].

It has been reported elsewhere [11] that the prevalence of RYO use is increasing in some countries. There is evidence that use has increased in the UK [12], and it has been argued that this is due to both the tax differential between RYO and FM in the UK and easy access to duty-free rolling tobacco in continental Europe [13]. To the extent that its cheaper cost is a prime motive, the Global Financial Crisis (GFC) could be driving any increases in RYO use identified in the study being reported here, especially in the USA and UK, where there was evidence of deteriorating economic conditions since 2005 [14–16] and where the impact has been particularly severe and long lasting.

In addition, industry documents reveal that the UK has been subject to a systematic campaign to change the image of RYO from a low-cost, down-market, product to a “cool,” “natural” choice [3]. On-pack advertising in Australia also reflects this strategy, and there is some anecdotal evidence that the myth that RYO tobacco is more “natural” (and by implication “safer”) is widespread in that country [17].

On the other hand, in Canada, the ease of access to cheaper contraband cigarettes [18] and the prevalence of discounting FM cigarettes are factors that would make the use of RYO for economic reasons less likely.

In an effort to extend the findings of our earlier work [3] based on data from the first wave of the ITC Four-Country Study, this study used six additional waves of data, a total of 7 waves covering the period from late 2002 to the end of 2008, specifically:

1. to examine trends in RYO use relative to FM cigarette smoking,
2. to determine if RYO prevalence has been rising in the UK and the USA, relative to Canada, given the different circumstances applying in those jurisdictions,
3. to examine whether RYO use was greater and/or has been increasing disproportionately among young, financially disadvantaged smokers, given the results of the NZ study,
4. to examine the prevalence of the reason that “RYO is less harmful” for smoking RYO and to determine if the importance of this reason has changed relative to other reasons for using RYO.

## 2. Methods

### 2.1. The ITC Project

The ITC Project is a multicountry study on tobacco use and tobacco control policy evaluation. Detailed descriptions of the project's conceptual framework and methods have been published elsewhere [19–21].

Participants were adult (18 years of age and older) cigarette smokers (who currently smoked at least once a month) from Canada, USA, UK, and Australia. The survey was designed as a longitudinal study to simultaneously evaluate several leading tobacco control policies subject to implementation over the time period of the study. The survey was conducted annually at around the same time of the year as much as possible with any variation in timing mainly for the purpose of enabling pre/posttests of policy changes (e.g., banning the term “lights” in the UK, labeling changes in Australia and Canada) [22]. The total number of participants was 19,456, a sample of approximately 2000 respondents per country per year (2002–2008), a retention rate of around 70% each year with 30% replenishment. Although ex-smokers are retained in the cohort, they are not included in the analyses reported here.

The survey field work was conducted using computer-assisted telephone interviews (CATIs). The survey was conducted in English or in French if desired in the Francophone areas of Canada. Strict protocols were developed and implemented to ensure equivalence of methods.

The study protocol was cleared for ethics by the Institutional Review Boards or Research Ethics Boards in each of the countries: the University of Waterloo (Canada), Roswell Park Cancer Institute (USA), University of Illinois-Chicago (USA), University of Strathclyde (UK), and The Cancer Council Victoria (Australia).

### 2.2. Measures

#### 2.2.1. RYO Use

All respondents were asked if they smoked “FM cigarettes only,” “mainly FM,” “FM and RYO similar,” “mainly RYO,” or “only RYO.” Based on these responses, RYO use was categorized in three ways: “Sometime RYO use” (mainly FM, FM & RYO similar); “Predominant RYO use” (mainly or only RYO, i.e., >50% of cigarettes smoked); and “Any RYO” use (i.e., either “sometime” or “predominant”).

#### 2.2.2. Sociodemographic Measures

Age (corrected for time in the sample), sex, income and education were measured the same way as previously reported [3, 17, 21]. From Wave 4 onwards smokers were also asked if they had been experiencing financial stress in the last 12 months (“unable to pay important bills on time”; yes/no), a single-item measure that has been used successfully in previous studies [23].

#### 2.2.3. Smoking Behaviors

They were heaviness of Smoking Index [24] (a combination of number of cigarettes per day with time to first cigarette), intention to quit (yes/no), and number of friends who smoke (out of a total of 5 closest friends).

#### 2.2.4. Reasons for Smoking RYO

This was a multiple response variable and has only been asked from Wave 5 onwards. Respondents were asked to identify up to four reasons from a list: because they are cheaper; because of the taste; because they help you reduce the amount smoked; because they are not as bad for your health.

### 2.3. Weighting and Statistical Analyses

All analyses were carried out using version 18.0.1 of the PASW (previously SPSS) statistical package. Weights have been designed to make the data representative of smokers in each of the four countries. There was no between-countries weighting. Weighted data are reported for the univariate and bivariate analyses, including self-reported prevalence. We used general estimating equations (GEEs) for multivariate analysis, since this technique allows for correlated data sets across the waves.

## 3. Results

### 3.1. Trends in the Prevalence of RYO Use

The prevalence of FM and RYO use by country across waves are presented in Table 1. The proportion of smokers using any RYO was highest in the UK and lowest in the USA in every wave. The prevalence of any RYO use relative to FM increased significantly in the UK (*P* < .001), while there was a nonsignificant increase in the USA (*P* = .078). It decreased significantly in Canada (*P* = .001) and marginally in Australia (albeit, not significantly; *P* = .055). These overall trends were supported by within-subjects data (i.e., data from those who were present across all 7 waves); there was more switching from exclusive FM to any RYO use in the UK, and the USA, and the reverse applied in Canada and Australia.

Over the study period, predominant use rose significantly in the UK and the USA, while there was a near-significant decline in Canada and Australia was flat. The prevalence of predominant RYO use as a proportion of any RYO use increased in all four countries (all *P* < .010).

### 3.2. Correlates of Predominant RYO Use

Because of the increasing relative and/or absolute prevalence of predominant use we decided to focus on predominant RYO use as a proportion of all cigarette use. The GEE analysis revealed that country was the variable most strongly associated with predominant use of RYO compared with all other forms of smoking (*P* < .001) (data not shown). There were also main effects of sex, income, heaviness of smoking, age, intention to quit (all *P* < .001), and number of smokers in their social network (*P* = .002). We also included the “financial stress” in the four Waves where it was measured, but it was not significant. We found significant interactions of country by sex, country by wave, country by age (all *P* < .001), and country by income (*P* = .007). Because of the strong by-country interactions, we carried out separate GEE analyses for each country (see Table 2).

The common correlates of predominant RYO use (compared with all other cigarette use) were *(low) income* and *(older) age*. However the age effect was weaker in Canada. Similarly, *males* reported more RYO use, but this trend was also smaller, and nonsignificant, in Canada. In the UK and Australia predominant users were significantly *less likely to intend to quit* than were other smokers. There was a similar trend in Canada, but not in the USA. In addition, predominant RYO users in Canada and Australia tended to be *heavier smokers*.

### 3.3. Comparison of Sometime Users with Predominant Users of RYO

A GEE analysis was carried out comparing sometime users with predominant users of RYO. The significant correlates of sometime use (rather than predominant use), using the seven waves of data were country, age, income, sex, (all *P* < .001), and wave (*P* = .019). A greater proportion of RYO smokers were sometime users in the USA (OR = 3.14; *P* < .001) compared with the UK (OR =  .57; *P* < .001), and compared with Wave 1, the relative prevalence of sometime use showed significant falls in Waves Four (OR =  .90; *P* = .047), Six (OR =  .86; *P* = .034) and Seven (OR =  .75; *P* < .001). Compared with predominant RYO users, sometime users were *more likely to have higher incomes* (OR = 1.27; *P* < .001) and, importantly, sometime users were *younger* than predominant RYO users, and the difference increased with age group (18–24 = reference, 25–39: OR =  .58; *P* < .001, 40–54: OR =  .39; *P* < .001, 55+ OR =  .29; *P* < .001). Compared with predominant users, they were also *less likely to be male *(OR =  .79; *P* < .001) and were marginally *more likely to intend to quit* (OR = 1.08; *P* = .054).

Given the large by-country interactions, results are presented separately by country. Sometime RYO smokers were *younger* than predominant RYO smokers in all four countries. In addition, Canadian sometime users smoked less, US sometime users were significantly less likely to be in the low income bracket, and UK and Australian sometime users were significantly less likely to be male. In addition, there was a significant interaction effect in Australia between age and wave with two clear age segments for sometime use emerging over the seven Waves (18–39 increasing prevalence and 40+ low prevalence).

**Figure 1:**
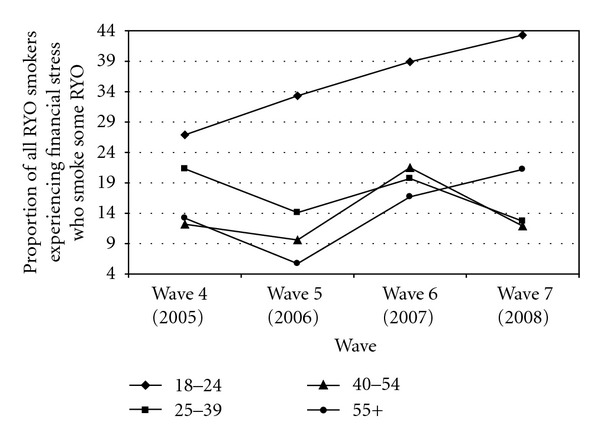
Proportion of all RYO users experiencing financial stress who smoke some RYO, by age group (4 country data, 2005–2008).

The relationship with “financial stress” was again tested using data from Waves 4–7. In this case, unlike the situation with respect to predominant use, significant interactions between age group and financial stress (*P* = .031) and wave and financial stress (*P* = .010) emerged.  Figure 1 shows the interaction between age and financial stress. This effect was independent of country, so we present the combined data. It is clear from the graph that young (18–24) smokers experiencing financial stress are not only disproportionate sometime RYO users across all four waves, and their level of sometime use has increased from 2005 (Wave 4). While those 55+ who are experiencing financial stress also show a rise in prevalence from Wave 5, their highest level of prevalence is lower than the lowest level of 18–24 year olds.

### 3.4. Reasons for Using RYO

The most common reason cited for using RYO (Table 3) was relative cost. From Waves 5 to 7, believing that RYO cigarettes are healthier increased significantly as a reason for using RYO in Canada (15.3% → 24.4%; *P* = .021), but no clear trend emerged in the other three countries. Australian RYO smokers identified health as a reason for smoking RYO more than RYO smokers from other countries. It is noteworthy that while those who predominantly use RYO, and those who are sometime users, give equal weight to saving money and the assumed health advantages, predominant RYO smokers are disproportionately inclined to cite “they taste better” as a reason for smoking RYO compared with sometimes users (64% versus 35%).

## 4. Discussion

We found the highest level of any RYO use is in the UK, followed by Australia, Canada and the USA, confirming and extending our earlier findings [3]. Consistent with our hypotheses, any use of RYO is increasing in the UK and probably in the USA, but is falling in Canada. RYO use relative to FM use is changing in quite different ways in the four countries under study, albeit with some core similarities. Understanding such a complex dynamic requires a systemic approach to the issue [25–29] to elucidate the dynamic relationships between countries, economic drivers, cultural norms, tobacco industry strategies, access to alternatives to RYO, tobacco control policies, and other factors.

Predominant use of RYO increased as a proportion of any RYO use in all four countries, most markedly in the USA, and increased as a proportion of total cigarette smoking in the USA and the UK. Compared to sometime RYO users, predominant users were more likely to have low-income, tended to be older, were disproportionately male and far more likely to cite “taste” as a reason for smoking RYO. However, young smokers experiencing financial stress were more likely to be sometime users than predominant users, and this interaction was independent of country.

We analysed the results to establish the extent to which they are consistent with price and financial need being the primary drivers of RYO use. Smokers themselves say that saving money is the main reason for RYO use, as this and other studies have found [1, 3, 5]. Further, use is highest in low income groups, especially predominant use. We assume that the typical pattern is for smokers to start using RYO on an occasional basis and only progress to predominant use if there are sufficient reasons for doing so (e.g., financial stress). Once this happens they begin to espouse different rationales for their RYO use (e.g., taste).

The clearest increases in predominant use were in the USA and UK: the two countries that arguably have been hardest hit by the GFC [14–16]. It is noteworthy that in Waves Six (2007) and Seven (2008), RYO smokers in the USA were more likely than FM smokers to say they were experiencing financial stress. One could speculate that in light of the financial pressures, in the USA smokers may have switched to RYO to reduce expenditure. The high, and increasing, level of UK RYO use reported by other studies [11, 12] was replicated. It is clear that RYO is a stable, mainstream market segment in the UK and easy access to duty-free RYO as well as a favourable tax regime [13] makes it relatively easy to reduce tobacco-related expenditure via RYO use.

Even before the GFC, the US industry was forecasting growth in the RYO segment, with cigarette manufacturers moving to take over existing niche manufacturers like Lane and Santa Fe. By 2004 Reynolds/Brown & Williamson thereby controlled 36% of the market, with Republic controlling an equivalent proportion [30]. Furthermore, as economic conditions deteriorated, manufacturers introduced tubes with longer filters (saving tobacco), and extra slim rolling papers, filter tips, and rolling machines [31].

The predominant use of RYO in Australia is relatively stable, but is increasing as a proportion of all RYO smoking, with use of sometime RYO falling substantially from Wave One to Wave Seven. The GFC affected Australia less than the USA and the UK and this may be partially responsible for the flat profile of predominant use compared to the clear increases in prevalence observed in the latter two countries.

The pattern of RYO use in Canada was the most distinct. Both predominant and sometime RYO use fell significantly (although sometime use fell proportionally more). The use of cheap contraband FM cigarettes among Canadians, especially the young [18], and the burgeoning share of discounted or cheap brands of cigarettes in that country, which had risen from 2% of the total market in 2002 to 42.8% in 2005 [32], are all factors that could help explain the decline. The net prevalence of RYO smokers (relative to FM smokers) saying they have been experiencing financial stress has been falling. It is likely that many of those experiencing substantial financial stress are using contraband tobacco or other low-cost alternatives that are available in Canada.

RYO cigarettes are an effective way of continuing to smoke at lower cost. This results in less revenue to government, made worse when the RYO tobacco is smuggled or otherwise taxed at lower rates. Of particular concern is the likelihood that this low-cost tobacco reduces incentives for smokers to quit. Similarly, there are concerns that RYO smoking might incur greater harm to health [7–9]. All these are good reasons for governments to act to reduce RYO use as part of an overall tobacco control strategy which could also include initiatives to support disadvantaged smokers (e.g., augmented programs of smoking cessation assistance and transfer of additional tax revenues to the poorest sectors of society).

Even though the proportion believing that RYO is healthier than FM use is a minority, that any group of smokers should hold such misconceptions is concerning. From a public health perspective, there is no justification for allowing tobacco companies to add “value” to RYO tobacco through messages about it being “natural” and “less harmful.”

In light of the prevalence of the health reason, we would argue that RYO smokers (especially the young) should not only be subject to the same health messages as other smokers but in addition, warnings on packaging and elsewhere should also stress that smoking RYO is at least as harmful as smoking FM. However, this needs to be qualified by the observation that peer-group pressure among young people is strong, and where a young peer group regularly uses RYO and reinforces use with myths about relative safety, health messages will need to be carefully framed. Clearly, research with such groups should be a prerequisite as part of adopting such a strategy.

Consideration also needs to be given to raising taxes on RYO to make its cost-point more comparable to FM cigarettes. This has been recommended previously [1] and a differentially higher one-off increase in excise tax on RYO tobacco has been imposed in New Zealand to help to stem rising RYO usage in that country (i.e., a tax of 24% on RYO versus 10% for FM cigarettes). This went some way toward equalizing RYO and FM cigarettes, but not entirely in terms of cost per cigarette, based on what is known about the weight of RYO cigarettes in NZ [4].

However, price-related interventions need to acknowledge that smokers will try to maximize the amount of nicotine they get from their delivery device, and research is needed to see if smokers respond by smoking their RYO cigarettes harder, something with the potential to increase harms. In considering tax equalization strategies, evidence from the UK that RYO tobacco is easier to smuggle means that such suggestions need to be carefully researched and backed up by increased resources to undermine trafficking of illicit tobacco.

Finally, trying to stop for-profit companies attempting to value-add to their products in search of increased sales is a futile exercise unless well-designed and enforced regulation is used. Governments need to confront the contradiction that allows companies to market products for profit that it is their avowed policy to discourage [33, 34] and do this for all forms of smoked tobacco. They should also do so in ways that minimize smuggling and other illicit supply, recognizing that this may be harder to stop for RYO tobacco than for FM cigarettes.

This study has several limitations. First, the relatively small number of RYO smokers in any given wave, especially in the USA, meant that following those smokers who stayed in the sample to monitor their choices of RYO versus FM cigarettes was impractical. In this situation the GEE technique enabled us to monitor aggregate changes in tobacco use and, at the same time, allow for interwave correlation. Second, it is extremely difficult, if not impossible, to quantify the links between exogenous drivers (e.g., the GFC, access to contraband, state/provincial tax regimes) using regression-based models like GEE.

## Figures and Tables

**Table 1:** Prevalence (%) of exclusive factory-made (FM) use, sometime (“Some”) RYO use, and predominant (“Pred”) RYO use by country and across waves (weighted data).

Wave (year)	Canada			United States			United Kingdom			Australia		
	FM	Some RYO	Pred RYO	FM	Some RYO	Pred RYO	FM	Some RYO	Pred RYO	FM	Some RYO	Pred RYO
1 (2002)	81.6	6.2	12.2	92.9	5.1	2.1	69.6	8.8	21.6	73.1	12.6	14.3
2 (2003)	83.0	5.9	11.5	93.4	4.4	2.3	68.2	7.4	24.4	75.2	9.9	14.9
3 (2004)	83.7	6.1	10.2	93.1	4.7	2.2	68.7	6.8	24.5	76.4	9.3	14.3
4 (2005)	83.9	5.1	11.0	91.2	5.9	2.8	67.6	6.2	26.2	77.5	7.8	14.7
5 (2006)	85.0	4.5	10.5	90.2	6.4	3.3	63.2	7.4	29.4	74.9	9.0	16.1
6 (2007)	87.3	4.2	8.6	90.3	4.5	5.2	62.3	6.1	31.5	77.3	7.7	15.0
7 (2008)	87.9	3.3	8.8	89.1	5.2	5.7	62.0	6.6	31.5	78.2	6.4	15.4

*P* value for trend	*.001*	*.006*	.080	.078	.677	*<.001*	*<.001*	*.039*	*<.001*	*.055*	*<.001*	.131

**Table 2:** Multivariate results of GEE analyses by country for predominant use of RYO compared to all other smoking patterns (factory-made cigarettes or “some” RYO).

	Canada			United States			United Kingdom			Australia		
	OR	CI	*P* value	OR	CI	*P* value	OR	CI	*P* value	OR	CI	*P* value
Wave (year)			*.005*			*.006*			*.054*			*.157*
1 (2002)	1.00	Reference		1.00	Reference		1.00	Reference		1.00	Reference	
2 (2003)	.99	.87–1.12	ns	1.10	.80–1.52	ns	1.04	.99–1.11	ns	1.06	.99–1.40	ns
3 (2004)	.99	.84–1.18	ns	1.12	.73–1.73	ns	1.07	.99–1.15	ns	1.09	.99–1.20	ns
4 (2005)	.97	.79–1.20	ns	1.17	.72–1.88	ns	1.09	.99–1.19	ns	1.04	.93–1.17	ns
5 (2006)	.79	.65–.97	.024	1.21	.66–2.22	ns	1.19	1.07–1.33	.001	1.12	.99–1.23	ns
6 (2007)	.59	.44–.79	<.001	2.08	1.37–3.15	.001	1.16	1.01–1.34	.035	1.05	.92–1.20	ns
7 (2008)	.57	.37–.87	.010	1.91	.87–4.20	ns	1.21	1.04–1.41	.014	1.10	.94–1.29	ns

Sex			*.875*			*.020*			*<.001*			*<.001*
Female	1.00	Reference		1.00	Reference		1.00	Reference		1.00	Reference	
Male	1.05	.61–1.83	ns	2.15	1.13–4.10	.020	2.93	2.53–3.38	<.001	1.93	1.64–2.28	<.001

Income			*<.001*			*<.001*			*<.001*			*<.001*
Low	1.00	Reference		1.00	Reference		1.00	Reference		1.00	Reference	
Medium	.64	.42–.99	.046	.46	.28–.78	.004	.89	.72–1.07	ns	.80	.70–.90	<.001
High	.16	.06–.42	<.001	.16	.09–.31	<.001	.70	.58–.84	<.001	.55	.46–.65	<.001

Age (years)			*.513*			*.173*			*.061*			*.005*
18–24	1.00	Reference		1.00	Reference		1.00	Reference		1.00	Reference	
25–39	1.07	.38–3.02	ns	1.47	.88–2.45	ns	1.57	1.00–2.50	.048	1.17	.94–1.46	ns
40–54	1.31	.42–4.02	ns	2.04	1.06–3.96	.034	1.64	1.07–2.53	.025	1.45	1.15–1.84	.002
55+	1.64	.52–5.16	ns	1.14	.57–2.28	ns	1.32	.86–2.04	ns	1.28	.98–1.68	ns

Intend to quit			*.356*			*.148*			*.022*			*.035*
No	1.00	Reference		1.00	Reference		1.00	Reference		1.00	Reference	
Yes	.88	.66–1.16	ns	2.01	.78–5.19	ns	.88	.79–.98	.022	.91	.83–.99	.035

HSI	1.26	1.12–1.4	<.001	.99	.86–1.16	ns	1.03	.99–1.08	ns	1.04	1.01–1.08	.019

No. of friends	.99	.90–1.07	ns	1.01	.87–1.16	ns	.99	.97–1.03	ns	1.02	.99–1.04	ns

*HIS: heaviness of smoking index, CI: 95% confidence interval, OR: adjusted odds ratio.

**Table 3:** Self-reported reasons for smoking RYO (all RYO users; Wave 7 in 2008, weighted data, multiple responses allowed).

Reason given	Percentage of respondents			
	Canada	US	UK	Australia
Cheaper than FM	93.2	94.3	95.4	85.4
Reduce amount smoked*	46.6	52.4	49.6	53.1
Taste	41.2	42.0	62.7	63.3
Healthier	24.5	28.3	26.9	39.6

*More specifically “because they help you reduce the amount smoked.”
